# Acute sleep deprivation-induced hepatotoxicity and dyslipidemia in middle-aged female rats and its amelioration by butanol extract of *Tinospora cordifolia*

**DOI:** 10.1186/s42826-024-00216-4

**Published:** 2024-08-21

**Authors:** Payal Bajaj, Tajpreet Kaur, Amrit Pal Singh, Gurcharan Kaur

**Affiliations:** 1 Medical Biotechnology Laboratory, Department of Biotechnology, Guru Nanak Dev University, Amritsar, 143005 Punjab India; 2 Department of Pharmacology, Khalsa College of Pharmacy, Amritsar, 143005 India; 3 Department of Pharmaceutical Sciences, Guru Nanak Dev University, Amritsar, 143005 India

**Keywords:** Sleep deprivation, *Tinospora cordifolia*, Hepatoprotection, Oxidative stress, Dyslipidemia, Apoptosis

## Abstract

**Background:**

Sleep deprivation (SD) due to an unhealthy lifestyle poses an oxidative challenge and is closely associated with an increased risk and prevalence of different metabolic disorders. Although the negative consequences of SD are well reported on mental health little is known about its detrimental effects on liver function and lipid metabolism. *Tinospora cordifolia* is reported for its hepatoprotective activity in different pre-clinical model systems. The current study was designed to elucidate the cumulative effects of aging and acute SD on liver functions, oxidative stress, and lipid metabolism, and their management by butanol extract of *T. cordifolia* (B-TCE) using middle-aged female acyclic rats as the model system.

**Results:**

Rats were divided into 4 groups: (1) Vehicle-undisturbed (VUD) (2) Vehicle-sleep deprived (VSD) (3) B-TCE pre-treated sleep-deprived (TSD) (4) B-TCE pre-treated undisturbed sleep (TUD). TSD and TUD groups were given 35 mg/kg of B-TCE once daily for 15 days followed by 12 h of sleep deprivation (6 a.m.–6 p.m.) of VSD and TSD group animals using the gentle-handling method while VUD and TUD group animals were left undisturbed. SD of VSD group animals increased oxidative stress, liver function disruption, and dyslipidemia which were ameliorated by B-TCE pre-treatment. Further, B-TCE was observed to target AMPK and its downstream lipid metabolism pathways as well as the p-Akt/cyclinD1/p-bad pathway of cell survival as possible underlying mechanisms of its hepatoprotective activity.

**Conclusions:**

These findings suggest that B-TCE being a multi-component extract may be a potential agent in curtailing sleep-related problems and preventing SD-associated hepatotoxicity and dyslipidemia in postmenopausal women.

**Graphical abstract:**

Graphical abstract to depict mechanism of action of B-TCE on liver function and lipid metabolism. 
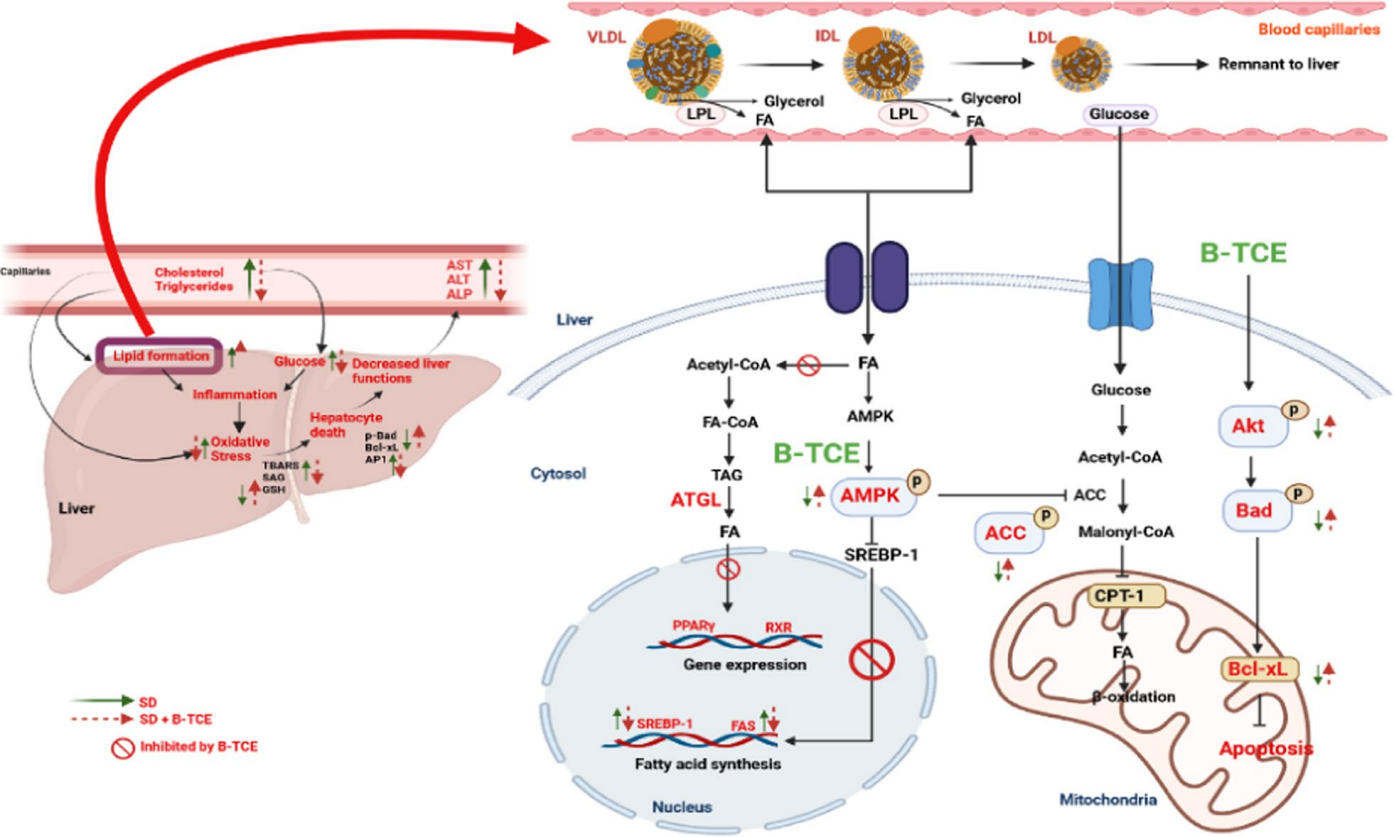

## Background

The middle-age in women’s aging trajectory is an important phase of their life for transitioning to healthspan in later life. Women undergoing midlife transition often, experience sleep problems, with some having occasional restless nights, whereas, others experience severe and chronic sleep disturbances that have a long-lasting negative impact on their health, quality of life, and ability to function in their day-to-day lives [1]. Moreover, sleep architecture is also independently influenced by aging besides hormonal changes. Current reports in the literature provide sufficient evidence that during times of hormonal transitions, the prevalence of sleep disturbances and sleep disorders increases, which is attributed to the key role of sex hormones in influencing sleep regulation [2]. Women, on the other hand, experience sleep differently in many ways from men mainly attributed to their biological life cycles of menstruation and menopause. Therefore, understanding sleep and its management in women is an important area of aging research.

Dysregulated metabolic activities adversely impact triglyceride, cholesterol, and fatty acid metabolism under sleep deprivation conditions [3] A meta-analysis that included 18 studies on 75,657 participants aged 18–96 reported that a short sleep duration of 5 h per day was associated with several metabolic disorders [4]. Sleep restriction has been found to increase insulin resistance in adipose tissue, which in turn results in inappropriate production of glucose in the liver and its reduced uptake by muscle consequently increasing free fatty acid release into the bloodstream [5]. Women’s lipid profile changes as they grow older and transit towards menopause, and these changes are frequently accompanied by increases in triglycerides, total cholesterol, and low-density lipoprotein cholesterol (LDL-C) [6, 7]. The intricate balance of lipid metabolism is further complicated by sleep deprivation in aging females. The effect of SD extends beyond neurological implications, as emerging evidence suggests that the level of hepatic cytokines and serum levels of aspartate aminotransferase (AST), alanine transaminase (ALT), and bilirubin increase in response to SD, resulting in liver damage [8, 9].

Rodents are widely used to model human menopause-related pathologies in view of their well-defined aging trajectories, and short life span of 2–3 years [10]. The majority of the studies on the menopause transition have used ovariectomized female rats as a model system. However, transitioning menopausal women rarely experience an abrupt reduction of ovarian steroids, as the post-menopausal ovary still secretes androgens and moderate amounts of other steroids [11] Since ovariectomy alters the HPG axis and hormonal profile much faster as compared to the intact reproductive tract [10], therefore, the selection of 13–15 months old acyclic female rats was considered appropriate to investigate the complex interplay between sleep deprivation, and menopausal transition, and their impact on lipid profile. Currently, available drugs that lower LDL levels and triglycerides while increasing High-density lipoproteins (HDL) levels such as statins, fibrates, and bile acid sequestrants are associated with the risk of developing cardiovascular diseases, increased insulin resistance, and the onset of type 2 diabetes [12–14]. Therefore, pre-treatment with B-TCE was tested to explore whether this natural dietary supplement can help alleviate the changes in liver functions during the menopause transition phase.

*T. cordifolia,* well reported for its hepatoprotective activity is known to prevent dyslipidemia and reduce serum AST, ALT, and ALP (Alkaline phosphatase) levels as reported in several pre-clinical studies [15, 16]. A clinical trial on hyperlipidemia patients who were on standard statin therapy was conducted using katuki (*Picrorhiza kurroa*) processed in *T. cordifolia* [17]. Serum levels of AST, ALT, and ALP were observed to improve significantly in the drug trial group patients as compared to the control group (placebo), thus suggesting the hepatoprotective potential of the plant extract. Similarly, the hepatoprotective efficacy of hepashrey syrup (a concoction of herbs including *T. cordifolia*) was tested in patients with hepatic disorders. Nine patients of both sexes were randomly selected on the basis of their hepatic complaints. The patients were administered 2½ teaspoons of syrup daily for 30 days [18]. Serum AST, ALT, and bilirubin levels were reduced significantly in hepashrey-treated patients in comparison to the control group suggesting hepatoprotective efficacy of hepashrey syrup. The amelioration of dyslipidemia and hyperglycemia by its different flavonoids is known to be mediated by AMPK activation [19]. Recently our lab furnished preliminary findings on the hepatoprotective potential of *T. cordifolia* stem powder (TCP) in high-fat diet-induced obesity. TCP was observed to prevent age-related metabolic and hepatic function impairment by targeting AMPK and its downstream pathway of lipid metabolism [20, 21]. Based on these previous lab reports, the current study was designed to investigate whether B-TCE, an active fraction of TCP, enriched with different phytochemicals, could alleviate SD-induced liver dysfunction, oxidative stress, and dyslipidemia in middle-aged female rats used as a perimenopausal model system. Further, the expression of key regulatory proteins involved in cell survival, apoptosis as well as lipid metabolism in the liver tissue was also studied.

## Methods

### Plant material

*T. cordifolia* (Willd.) Miers, stems growing on multiple Neem (*Azadirachta indica*) trees were harvested in the month of May (35–40 °C) from a forest situated in the Ropar district of Punjab, India. The specimen was authenticated by a taxonomist and a voucher sample (Accession No. 65 Bot. & Env. Sc. dated 04-09 2017) was deposited at the herbarium in the Department of Botanical and Environmental Sciences, GNDU, Amritsar, India for future reference.

### Preparation of extract

*T. cordifolia* stems were washed thoroughly with tap water, kept in a hot air oven at 45 °C for 10–15 days until completely dried, and then grounded to a fine powder. The powdered stem (1 kg) was immersed in 50% ethanol and then extracted following the percolation method and then filtered using Whatman filter paper No. 1. Rota-vapor (Buchi R-210, Switzerland) was used to concentrate the extract under reduced pressure at 40 °C to yield 108 g of crude ethanolic extract (TCE). TCE was dissolved in water and further fractionated in different organic solvents with increasing polarity such as (a) hexane, (b) chloroform, (c) ethyl acetate, and (d) butanol (SRL, Analytical grade, 95% purity) to yield 0.245 g of hexane, 5.23 g of chloroform, 3.833 g of ethyl acetate and 13.25 g of n-butanol fractions. The fractions thus obtained were concentrated in vacuo in a rotary evaporator. n-Butanol extract (B-TCE) obtained was used for further studies. The dose of B-TCE (35 mg/kg) for the current study has been previously standardized in our lab [22].

### Experimental design

Adult female Wistar strain rats, 13–15 months old and weighing about 200-250 g were selected for the current study. After a week of acclimatization, animals were housed 3 rats per cage under controlled environmental conditions (25 ± 2 °C, 50% humidity) and 12 h light/12 h dark cycle. The animals were provided ad libitum access to water and food. The experiments were approved and performed in accordance with the guidelines of the Institutional animal ethical committee (IAEC) of GNDU, Amritsar, India (Reference no: 226/CPCSEA/2019/10).

The animals were divided into 4 groups with n = 6–7 rats in each group.

Group I: Vehicle-undisturbed group (VUD).

Group II- Vehicle-sleep deprived (VSD).

Group III- B-TCE (35 mg/kg) pre-treated sleep-deprived group (TSD).

Group IV- B-TCE (35 mg/kg) pre-treated undisturbed sleep group (TUD).

Animals of the TSD and TUD group were orally gavaged with 35 mg/kg of B-TCE between 9:00–10:00 am daily for 15 consecutive days, whereas, VUD and VSD group animals were given an equal volume of water as vehicle. The gentle handling method was employed to sleep-deprive VSD and TSD group animals for 12 h on the 15th day (from 6 am-6 pm) while VUD and TUD group animals were left undisturbed. The gentle handling method involves depriving animals of sleep either by gently shaking their cages or by stroking them with a soft brush with minimal disturbance.

### Serum and liver markers

Following SD, animals in different groups were anesthetized with sodium thiopentone injection (1 unit/10 g body weight, intraperitoneal). Blood samples were collected through cardiac puncture and animals were sacrificed by cervical dislocation. Blood was allowed to clot at room temperature and centrifuged at 10,000 rpm for 15 min to separate serum, which was later used for the estimation of different serum parameters using commercially available kits (ERBA Mannheim kits, Transasia Bio-medicals Ltd, India) as per the manufacturer’s instructions.

#### AST estimation

For AST estimation (Cat no- 120204), 1000 µL of AST reagent (Mixture of 12 mmol/L of 2-oxoglutarate, 200 mmol/L of L-Aspartate, 545 U/L of MDH, 909 U/L of LDH, 0.18 mmol/L of NADH, 80 mmol/L of Tris buffer and 5 mmol/L of EDTA) was mixed with 100 µL of blank, standard and test solutions respectively in separate vials followed by incubation at 37 °C for 1 min. The change in absorbance at different time intervals of 1, 2 and 3 min was then measured at 340 nm [23].

#### ALT estimation

For ALT estimation (Cat no-120207), 1000 µL of ALT reagent (Mixture of 137.5 mmol/L of Tris buffer, 2000 U/L of LDH, 7.9 mmol/L of L-Alanine, 20 mmol/L of CAPSO, 85 mmol/L of 2-oxoglutarate, and 1.05 mmol/L of NADH) was mixed with 100 µL of blank, standard and test solutions respectively in separate vials followed by incubation at 37 °C for 1 min. The change in absorbance at different time intervals of 1, 2 and 3 min was then measured at 340 nm [23].

#### ALP estimation

For ALP estimation (Cat no-120247), 1000 µL of ALP reagent (Mixture of 435 mmol/L of AMP buffer, 1.24 mmol/L of Zinc sulfate, 2.48 mmol/L of Magnesium acetate, 2.48 mmol/L of HEDTA and 19.5 mmol/L of p-nitrophenyl phosphate) was mixed with 100 µL of blank, standard and test solutions respectively in separate vials followed by incubation at 37 °C for 1 min. The change in absorbance at different time intervals of 1, 2 and 3 min was then measured at 340 nm [23].

#### Glucose estimation

For serum glucose (Cat no-120200) estimation, 1000 µL of working reagent (mixture of 20,000 IU/L of glucose oxidase, 3250 IU/L of Peroxidase, 10 mmol/L of 4-hydroxybenzoic acid, 0.52 mmol/L of 4-Aminoantipyrine, and 110 mmol/L of phosphate buffer) was mixed with 10 µL of blank, standard and test respectively in a separate vial. The mixture was then incubated at 37 °C for 15 min followed by measurement of absorbance at 505 nm [24].

#### Cholesterol estimation

For cholesterol (Cat no-120194) levels estimation, 1000 µL of working reagent (mixture of 200 IU/l of cholesterol esterase, 150 IU/l of cholesterol oxidase, 20 mmol/l of sodium phenolate, 2000 IU/l of HRP, 0.5 mmol/l of 4-aminoantipyrine, and 68 mmol/l of phosphate buffer) was mixed with 20 µL of blank, standard and test respectively in a separate vial. The mixture was then incubated at 37 °C for 10 min followed by measurement of absorbance at 505 nm [24].

#### Triglyceride estimation

For estimation of triglycerides (Cat no-120211) levels, 1000 µL of working reagent (mixture of 2.5 mmol/l of ATP, 0.8 mmol/l of 4-Aminoantipyrine, 2.5 mmol/l of Mg^2+^, 3,5 DHBS, 550 U/l of glycerol kinase, 2000 U/l of peroxidase, 8000 U/l of GPO, 3500 U/l of lipoprotein lipase and 53 mmol of buffer) was mixed with 10 µL of blank, standard and test respectively in a separate vial. The mixture was then incubated at 37 °C for 10 min followed by measurement of absorbance at 505 nm [24].

### Biochemical quantification of oxidative stress markers

To estimate oxidative stress markers, the liver tissue was rinsed with a 1.17% potassium chloride (KCl) solution. A small part of the tissue was used for superoxide anion generation (SAG) estimation using the method described in the later section whereas, the rest of the tissue was homogenized using a Teflon homogenizer in 1.17% KCl (10% w/v) solution. The homogenate was centrifuged for 20 min at 800 × g in order to remove cellular debris and then recentrifuged for 10 min at 11,000 × g at 4 °C. The supernatant obtained was used to estimate reduced glutathione and thiobarbituric acid reactive substances (TBARS) levels. For TBARS levels, 2 mL of TBA solution containing 15% trichloroacetic acid (TCA), 0.375% thiobarbituric acid (TBA) and 0.25N hydrochloric acid (HCl) was added to 1 mL of liver homogenate and mixed thoroughly, followed by incubation in boiling water bath for 15 min. After cooling, samples were centrifuged for 10 min at 10,000 × *g* resulting in color formation which was measured spectrophotometrically at 535 nm. 1,1,3,3 tetramethoxy propane was used as a standard in the range of 1–10 nM and was processed in the same manner. Results are expressed as nanomoles per milligram of protein (nM/mg of protein). For reduced glutathione quantification, 1 mL of the liver homogenate was mixed with trichloroacetic acid (10% w/v) and centrifuged. It was then followed by the addition of 0.25 mL of 0.001 M Ellman’s reagent (5’- dithiobis- [2-nitrobenzoic acid]) in clear supernatant and absorbance of the solution was measured at 412 nm. A standard plot using GSH (10–100 μM) was plotted and results were expressed as micromoles of reduced glutathione per milligram of protein (μM of GSH/mg of protein) [23].

#### Superoxide anion generation

25 mg of liver tissue was added in 5 mL of phosphate-buffered saline (PBS) containing 100 µM of nitroblue tetrazolium (NBT) followed by incubation at 37˚C for 90 min. The reaction was stopped by adding 5 mL of 0.5 M HCl. The liver tissue was then removed and homogenized in 1 mL of the mixture containing 0.1 M sodium hydroxide (NaOH) and 0.1% sodium lauryl sulfate (SLS) in water containing 40 mg/L of diethylenetriaminepentaacetic acid (DTPA) and then centrifuged for 20 min at 20,000 × *g*. The supernatant was decanted and the pellet was resuspended in 1.5 mL of pyridine and was left undisturbed for 90 min at 80 °C to extract formazan, an adduct formed after reaction of NBT with superoxide anions. The mixture was again centrifuged for 10 min at 10,000 × *g* and the absorbance of formazan product was determined spectrophotometrically at 540 nm [23].

### Histological study of liver tissue

Excised liver tissues stored in 10% neutral buffered formalin were dehydrated in a graded concentration of alcohol, immersed in xylene, and embedded in paraffin. 5 µm thick sections were cut using a microtome and stained with hematoxylin–eosin stain (H & E) using the method described in [22].

### Western blotting

Liver tissue (n = 6 per group) was lysed in a lysis buffer (DTT, Na_3_VO_4_, 1X Tris-buffered saline, and Protease inhibitor cocktail) using a tissue homogenizer. The homogenate was centrifuged at 8000 rpm for 15 min at 4 °C and the supernatant obtained was assayed for total protein concentration using the Bradford dye binding assay. Bradford method determines the concentration of protein by measuring the change in color of the samples on reacting with Coomassie Brillant Blue (CBB) G-250 dye (Sigma Aldrich, CAS 6104-58-1) caused by aromatic amino acids lysine, arginine, and histidine. The density of the color obtained is proportional to the protein concentration. A standard curve was prepared using 190 µL of Bradford reagent added to 10 µL of bovine serum albumin (BSA, Himedia Laboratories GRM3151-100G) in the range of 100 µg to 1 mg/mL. After mixing, the absorbance of the samples was measured at 450 nm and 595 nm using a spectrophotometer. The protein concentration was calculated by comparing the absorbance of samples to the BSA standard curve. 50 µg of protein was then solubilized in 6X SDS-sample buffer and heated in boiling water for 2 min and then separated on 8–10% SDS-PAGE (60–80 V) and transferred (100 V, 2 h) onto a 0.45 µm PVDF membrane (Hybond-P, Amersham Biosciences, UK) using a wet transfer system (Mini-PROTEAN Tetra Cell, Bio-Rad Laboratories Inc, USA). The membranes were then blocked for 2 h with 5% skim milk powder in 0.1% Tris-buffered saline Tween 20 solution (TBST) and incubated overnight at 4˚C with appropriate mouse monoclonal antibodies.

Primary antibodies such as: 1:1000 dilution of rabbit anti-p-AMPKα, rabbit anti-AMPKα, mouse anti-p-ACC, mouse anti-ACC (AMPK and ACC sampler kit #9957) (all from Cell Signaling Technology, USA), rabbit anti-SREBP-1 (Cat no-SAB4502850), rabbit anti-FAS (Cat no- F9554), rabbit anti-HMG-CoAR (Cat no- ABS229), and rabbit anti-ATGL (Cat no- SAB5702559), 1: 1500 dilution of rabbit anti-p-bad (Cat no- SAB5700282), and rabbit anti-AP1 (Cat no- A5968), 1:2000 dilution of rabbit anti-p-Akt (Cat no- SAB4504331), and mouse anti-Bcl-xL (Cat no- B9429), rabbit anti-pan-Akt (1:3000) (Cat No- SAB4301170), mouse anti-cyclinD1 (1:2500) (Cat no- C7464), (all from Sigma-Aldrich, USA), rabbit anti-eNOS (1:2000) (Cat no- sc136977) (Santa Cruz Biotechnology, USA) were used overnight at 4˚C. Mouse anti-α-tubulin (1:5000) (Cat no- T6199) (Sigma-Aldrich, USA) was used as an endogenous loading control. The membranes were washed thrice with 0.1% TBST (tris-buffered saline and tween-20) for 10 min and subsequently incubated (2 h, 25˚C) with specific HRP-conjugated secondary antibodies (GeNei, India). Following this, membranes were washed three times with 0.1% TBST (10 min each), and protein bands were visualized by adding Amersham ECL reagent using the ImageQuant LAS 4000 system. AlphaEase FC software (Alpha Innotech CA, USA) was used for analyzing the change in the protein expression.

### Densitometry and statistical analysis

For the quantification of protein bands, the Spot Denso tool of AlphaEase FC software was used which gave integrated density values (IDV) of each protein band. The value of each protein band was then normalized to the IDV value obtained from its corresponding loading control band i.e. α-tubulin. The quantified densitometry was then presented in a bar graph and is expressed as a percentage of control. GraphPad Prism software, Version 8 was used for statistical analysis. All the data are expressed as mean ± SEM and were compared using two-way ANOVA followed by Tukey’s post hoc method considering sleep deprivation and B-TCE as two independent variables in the study. Values with *p* ≤ 0.05 were considered as a threshold for significance.

## Results

### B-TCE ameliorated SD-induced liver function impairments

**Fig. 1:**
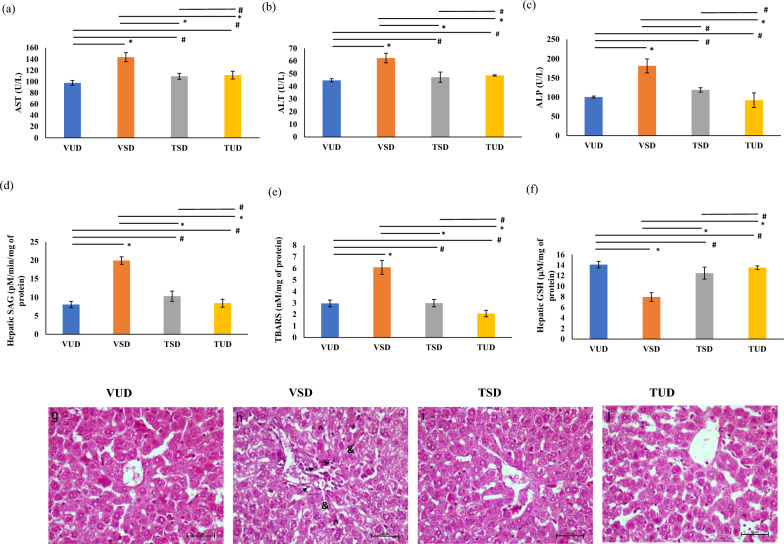
B-TCE Supplementation of B-TCE attenuated SD induced liver function impairments and oxidative stress. **a**, **b**, **c** Levels of hepatic enzymes, AST, ALT, and ALP were significantly reduced in the serum of TSD and TUD group animals in comparison to the VSD group. **d**, **e** Hepatic SAG and TBARS levels increased significantly in the VSD group which were normalized by B-TCE pre-treatment in TSD and TUD group animals. **f** Hepatic GSH level which was reduced significantly in VSD group animals was restored in TSD and TUD groups with B-TCE supplementation. **g**, **h**, **i**, **j** H & E staining of hepatic tissue in VUD, VSD, TSD, and TUD group animals (200X magnification) where arrows indicate inflammatory cells, ‘&’ represents congestion and ‘^’ represents necrosis. Statistically significant difference between groups was obtained using two-way ANOVA analysis followed by Tukey’s post-hoc test and is indicated as follows: ^*^ represents *p* ≤ 0.05 which signifies statistically significant difference between different group animals, and # represents *p* ≥ 0.05 which signifies non-significant difference between different group animals

Liver function markers were tested to assess the toxicity if any, of B-TCE pre-treatment and also to elucidate its potential beneficial effects to mitigate SD-induced alterations in liver tissue. A significant increase (*p* ≤ 0.05) in the serum levels of AST, ALT, and ALP from VSD group animals suggests acute SD-induced liver dysfunction (Fig. 1a, b, c). Pre-treatment with B-TCE in TSD and TUD group animals significantly suppressed (*p* ≤ 0.05) these changes and their levels were comparable to the VUD group animals (Fig. 1a, b, c) thus suggesting the hepatoprotective potential of B-TCE in acute SD rats.

### B-TCE attenuated SD-induced oxidative stress and histological changes

A significant increase (*p* ≤ 0.01) in the concentration of oxidative stress markers SAG and TBARS was observed in the VSD animals in comparison to the VUD group, which were normalized by B-TCE pre-treatment in TSD and TUD group animals (Fig. 1d, e). On the other hand, the level of antioxidant, reduced glutathione (GSH) which was significantly reduced (*p* ≤ 0.001) in the VSD group as compared to the VUD animals, was significantly recovered (*p* ≤ 0.05) by prior treatment with B-TCE in both TSD and TUD group animals (Fig. 1f). Further, H & E staining of hepatic tissues in the VSD group revealed congestion, inflammation, and necrosis as compared to the VUD group. B-TCE pre-treatment ameliorated SD-induced hepatic changes in TSD rats, whereas, extract alone in the TUD group did not alter liver architecture (Fig. 1g, h, i, j).

### B-TCE pre-treatment prevented SD-induced changes in blood glucose, cholesterol, and triglyceride levels

**Fig. 2:**
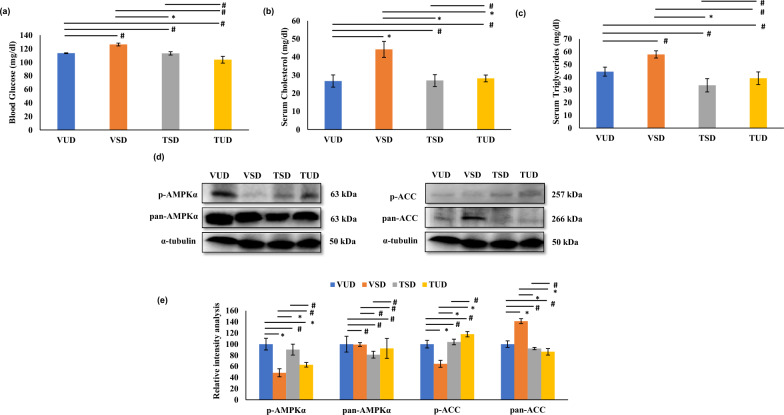
B-TCE supplementation-maintained serum lipid profile and prevented dyslipidemia. **a**, **b**, **c** Histograms represent a significant reduction in the levels of serum glucose, cholesterol, and triglycerides in TSD and TUD group animals as compared to the VSD group. **d** Representative western blot images (n = 6 per group) of p-AMPKα, AMPK, p-ACC, and pan-ACC in VUD, VSD, TSD, and TUD group animals. **e** Densitometry was used to analyse the immunoreactive bands and the resulting histograms represent percentage change in the expression of p-AMPKα, AMPK, p-ACC, and pan-ACC in the liver tissue of different group animals. Statistically significant difference between groups was obtained using two-way ANOVA analysis followed by Tukey’s post-hoc test and is indicated as follows: ^*^ represents *p* ≤ 0.05 which signifies statistically significant difference between different group animals, and # represents *p* ≥ 0.05 which signifies non-significant difference between different group animals

Hypercholesterolemia and hypertriglyceridemia are metabolic conditions marked by elevated cholesterol and triglyceride levels in the blood. SD for 12 h resulted in a significant increase (*p* ≤ 0.05) in blood cholesterol levels and a marginal increase in blood glucose and triglyceride levels in VSD group animals in contrast to the VUD group (Fig. 2a, b, c). However, pre-treatment with B-TCE prior to SD resulted in significant downregulation (*p* ≤ 0.05) in the level of glucose, cholesterol, and triglyceride in TSD group animals in comparison to the VSD group rats. The level of these metabolites in the TUD group was similar to VUD rats (Fig. 2a, b, c).

### B-TCE modulated the expression of proteins involved in lipid metabolism

**Fig. 3:**
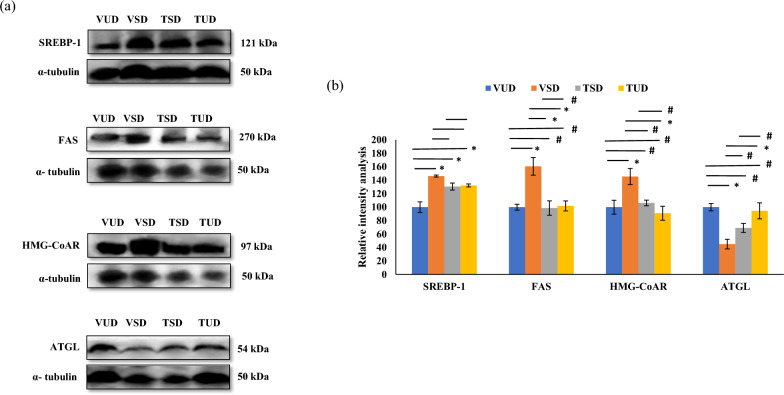
Effect of B-TCE pre-treatment on the expression of proteins involved in lipogenesis (SREBP-1 and FAS), cholesterol biosynthesis (HMG-CoAR), and lipolysis (ATGL) in sleep-deprived rats. **a**, **b** Representative western blot images (n = 6 per group) and densitometric analysis representing fold change in the expression of SREBP-1, FAS, HMG-CoAR, and ATGL in the liver tissue of different group animals. Statistically significant difference between groups was obtained using two-way ANOVA analysis followed by Tukey’s post-hoc test and is indicated as follows: ^*^ represents *p* ≤ 0.05 which signifies statistically significant difference between different group animals, and # represents *p* ≥ 0.05 which signifies non-significant difference between different group animals

To further understand the underlying molecular mechanism(s) of hepatoprotection and correction of dyslipidemia by B-TCE, the expression of proteins associated with lipid metabolism such as AMPK, ACC, SREBP-1, FAS, HMG-CoAR, and ATGL were studied in the liver tissue. A significant decrease (*p* ≤ 0.01) in the expression of p-AMPKα and p-ACC was observed in VSD animals in comparison to the VUD group and these changes were restored significantly (*p* ≤ 0.05) by B-TCE supplementation in TSD and TUD group rats (Fig. 2d, e). No significant change was observed in the expression of pan-AMPKα among different group animals. However, in comparison to VUD animals, a significant upregulation (*p* ≤ 0.01) in the expression of pan-ACC was observed in VSD animals, which was significantly downregulated (*p* ≤ 0.05) by pre-treatment with B-TCE in both TSD and TUD group animals (Fig. 2d, e). To further identify the targets of lipid biosynthesis and lipolysis by B-TCE, the expression of proteins involved in regulating fatty acid synthesis such as SREBP-1, FAS, lipolysis, ATGL (Adipose triglyceride lipase), and cholesterol biosynthesis such as HMG-CoAR (3-hydroxy-3-methylglutaryl-coenzyme A) were studied (Fig. 3a, b). Acute SD resulted in significant upregulation (*p* ≤ 0.01) in the expression of lipogenic enzymes, SREBP-1, and FAS as compared to the VUD group animals. B-TCE pre-treatment significantly suppressed (*p* ≤ 0.01) these changes in FAS expression (Fig. 3a, b). Although, B-TCE supplementation downregulated the expression of SREBP-1 in the TSD group animals but the change was not found to be statistically significant. In contrast, B-TCE pre-treatment upregulated the expression of lipolytic enzyme, ATGL as compared to the VSD group (Fig. 3a, b). Furthermore, cholesterol synthesis regulating protein HMG-CoAR expression was also significantly upregulated (*p* ≤ 0.05) in the VSD rats in contrast to the VUD group, and B-TCE pre-treatment effectively suppressed these changes in both TSD and TUD rats (Fig. 3a, b). These findings are suggestive of the potential beneficial effects of B-TCE in preventing dyslipidemia induced by SD.

### B-TCE provided hepatoprotection by upregulating the cell survival p-Akt pathway

**Fig. 4:**
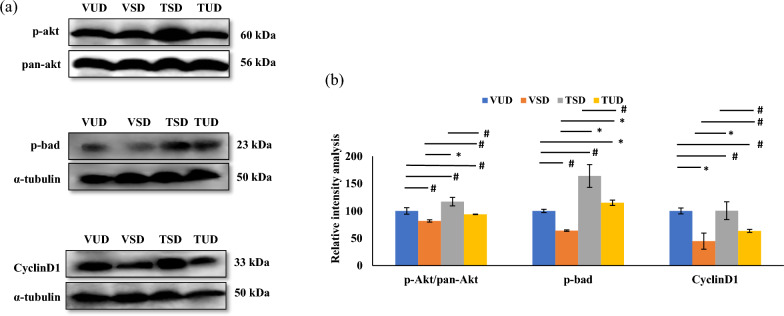
Cell growth promotion by B-TCE. **a**, **b** Representative western blot images (n = 6 per group) and densitometric analysis representing fold change in the expression of p-Akt, p-bad, and cyclinD1 in the liver tissue of different group animals. Statistically significant difference between groups was obtained using two-way ANOVA analysis followed by Tukey’s post-hoc test and is indicated as follows: ^*^ represents *p* ≤ 0.05 which signifies statistically significant difference between different group animals, and # represents *p* ≥ 0.05 which signifies non-significant difference between different group animals

**Fig. 5:**
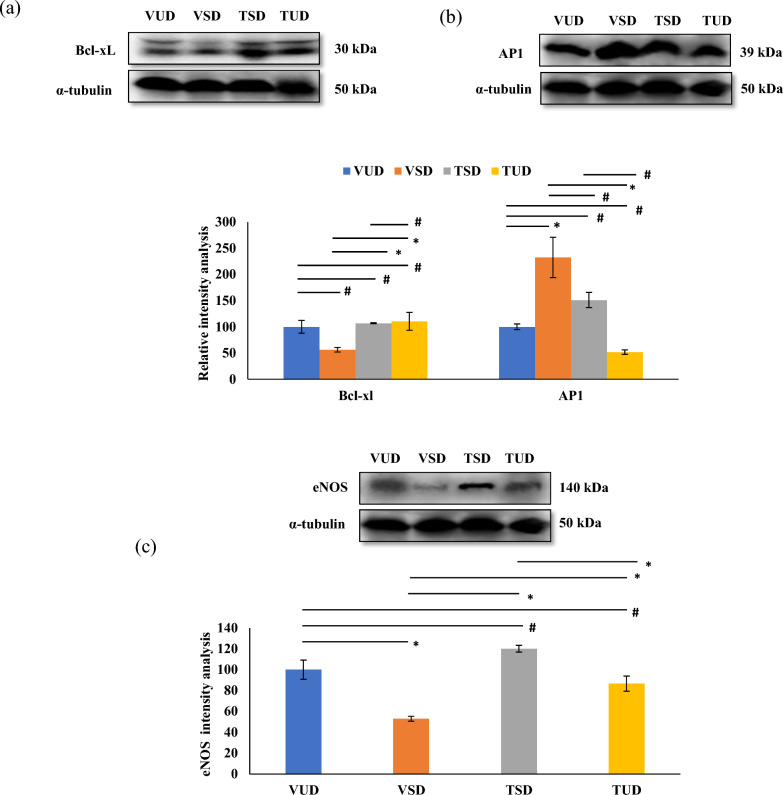
Inhibition of apoptosis and maintenance of endothelial functions by B-TCE. **a**, **b**, **c** Representative western blot images (n = 6 per group) and densitometric analysis representing fold change in the expression of Bcl-xL, AP1, and eNOS in the liver tissue of different group animals. Statistically significant difference between groups was obtained using two-way ANOVA analysis followed by Tukey’s post-hoc test and is indicated as follows: ^*^ represents *p* ≤ 0.05 which signifies statistically significant difference between different group animals, and # represents *p* ≥ 0.05 which signifies non-significant difference between different group animals

The expression of proteins implicated in cell proliferation and apoptosis such as p-Akt, p-Bad, cyclin D1, Bcl-xL, and AP1 were further studied. As compared to the VUD group, sleep-deprived rats showed reduced expression of p-Akt/pan-Akt, cyclin D1, p-bad, and Bcl-xl in VSD group animals (Figs. 4a, b and 5a). These changes were significantly (*p* ≤ 0.05) suppressed in the B-TCE pre-treated group. However, the expression of an apoptotic protein, AP1 (Activator protein-1) was increased significantly (*p* ≤ 0.01) in the VSD group in comparison to the VUD group animals which was alleviated by pre-treatment with B-TCE (Fig. 5b). Furthermore, expression of e-NOS (Endothelial-nitric oxide synthase), marker regulating endothelial function was significantly downregulated (*p* ≤ 0.01) in the VSD group, and this change was suppressed by B-TCE supplementation in TSD and TUD group (Fig. 5c).

## Discussion

Sleep disturbances affect about 40–60% of peri- and post-menopausal women. NIH State-of-the-Science Conference (2005) statement cited sleep disturbances as a key symptom of menopausal transition [25]. Deterioration of sleep quality such as increased bouts of awakenings, and sleep fragmentation starts in the perimenopausal period. A longitudinal study over a span of 5 years on premenopausal women also attributed poor sleep quality in women during perimenopause to daytime sleepiness, underlying depressive symptoms, and use of CNS-active medications [26]. Chronic sleep loss is reported in 31–42% of women by the time they are through their menopausal transition [27].

Currently available hormone replacement therapy and drugs such as statins, fibrates, and bile acid sequestrants for the management of lipid metabolism in sleep-deprived middle-aged menopausal women are associated with severe side effects such as hepatotoxicity, inflammation, myopathy, CNS disorders, etc. [28–30]. This necessitates the need to explore natural compound-based therapeutic agents which are safe and effective alternatives for modulating liver lipid metabolism in aged peri-menopausal women. *T. cordifolia* widely known as “Guduchi” or “Gilloy” is a deciduous shrub native to India. Extracts of this plant have been reported to possess immunomodulatory**,** anti-osteoporotic, anti-diabetic, anti-arthritic, anti-cancer, hepatoprotective, and anti-oxidant activities [31]. Previous studies from our lab have reported the neuroprotective potential of different extracts of *T. cordifolia* in different animal model systems [32, 33]. Butanol extract of *T. cordifolia* (B-TCE) was also observed to possess neuroregenerative and neuroprotective potential against glutamate-mediated excitotoxicity tested under both in vitro and in vivo conditions [22, 34]. Recently, we reported that B-TCE supplementation alleviates motor dysfunctions and cognitive deficits associated with acute SD at a concentration of 35 mg/kg, [35] which was much lower than 140 mg/kg of parent 50% ethanolic extract (TCE) [32]. The current study was designed to explore the potential hepatoprotective activity of B-TCE against acute SD-induced hepatotoxicity and dyslipidemia in middle-aged female rats as a model of the midlife transitional period.

A significant increase observed in the serum levels of AST, ALT, and ALP in VSD animals suggests that acute SD during the light phase may cause liver dysfunction. ALT and AST are biochemical markers that determine the structural and functional integrity of the liver and elevated levels of these enzymes denote the initiation of cellular damage [36]. VSD group animals showed significantly higher levels of serum ALT and AST which were normalized by B-TCE supplementation thus suggesting the hepatoprotective potential of B-TCE (Fig. 1a, b). Previous animal studies have also reported an increase in serum AST and ALT levels in response to SD [36, 37]. ALP, an extracellular hydrolytic enzyme, is considered a cholestasis induction biomarker [38]. The increased ALP level in the VSD group may be suggestive of biliary tract dysfunction induced by SD. However, a significant reduction in ALP level in B-TCE pre-treated group provides evidence of hepatoprotective activity of B-TCE against SD-induced toxicity (Fig. 1c). The current findings are supported by the previous reports of hepatoprotection by *T. cordifolia* extracts against paracetamol and carbon tetrachloride-induced toxicity by reducing the levels of serum AST, ALT, and ALP in the different animal model systems [15, 39]. Recently, ethanolic extract of *T. cordifolia* has been reported to mitigate titanium dioxide nanoparticle-induced hepatotoxicity in *Oreochromis niloticus* by decreasing levels of serum AST, ALT, and ALP [40]. *T. cordifolia* was also observed to mitigate the effect of chronic and moderate alcohol intake on liver and intestinal absorption by reducing the levels of serum AST, ALT, and ALP [41]. The data of TSD and TUD group animals suggests that the dose of B-TCE used was not toxic and rather showed hepatoprotective activity against acute SD-induced liver function impairment.

Acute SD-induced oxidative stress is evident from an increase in hepatic TBARS, SAG, and reduced GSH levels in VSD group animals (Fig. 1d, e, f). An imbalance between ROS synthesis and antioxidant activity leads to oxidative stress, impacting signal transduction and cellular resilience, primarily by stimulating membrane lipid peroxidation and oxidation of genes and proteins [42, 43]. Excessive ROS formation can disrupt the mitochondrial membrane, and this mitochondrial dysfunction further stimulates ROS production. ROS are accumulated during wakefulness while sleep promotes its removal by stimulating the antioxidative machinery in the liver tissue which is the principal detoxifier responsible for eliminating ROS from the body. However, the liver’s capacity to remove excess ROS is compromised by SD [44]. Superoxide anion is a prominent ROS species which mediates various oxidative chain reactions and is also a precursor to different ROS species. An investigation of intracellular superoxide levels can therefore provide an estimate of intracellular ROS levels [45]. The most extensively used marker of oxidative stress detection in clinical studies is the assessment of malondialdehyde (MDA), an end product of lipid peroxidation through TBARS (Thiobarbituric reactive substances) levels [42]. Plasma lipid peroxidation is found to be significantly higher after ischemic stroke as well as in patients with atherosclerosis [46, 47]. Since ROS reacts with membrane phospholipids to cause lipid peroxidation, [48] supporting the marked increase observed in superoxide anion generation (SAG) and TBARS levels in the VSD group animals (Fig. 1d, e, f). Several previous reports support these findings of SD-mediated increase in serum TBARS levels [49, 50]. In contrast, Glutathione is one of the key molecules necessary for maintaining the integrity of the cell membrane against alkylating and oxidating agents [51]. Reduced glutathione (GSH) levels below its basal level stimulate ROS production, exposing mitochondria to its own endogenously synthesized free radicals, resulting in irreversible damage and affecting the structural and functional integrity of the membrane [52].The current data suggest that B-TCE supplementation inhibited lipid peroxidation and superoxide anion generation by promoting intracellular antioxidative machinery as evidenced by reduced TBARS and SAG and high GSH levels in TSD and TUD group animals (Fig. 1d, e, f). These results are consistent with a previous report where *T. cordifolia* supplementation was found to reduce TBARS and increase GSH levels in the liver of experimentally induced type 2 diabetic rats [53]. Results are further supported by H & E staining that revealed hepatic damage in the VSD group and its correction with B-TCE supplementation (Fig. 1g, h, i, j).

SD has been observed to induce lipogenesis in the rat liver which is more pronounced in females than the males [54, 55]. Hyperlipidemia is a major risk factor for CVDs which is characterized by an increase in the levels of serum triglycerides, cholesterol, and low-density lipoprotein cholesterol (LDL) [56]. Consistent with these earlier studies, the current data shows that sleep-deprived animals had higher serum levels of glucose, triglyceride, and cholesterol than their age-matched VUD group, and these changes were attenuated by B-TCE pre-treatment in both TSD and TUD group thus providing further evidence of B-TCE as a potential candidate to manage SD-associated dyslipidemia (Fig. 2a, b, c). *T. cordifolia* supplementation has also been earlier reported to reduce serum cholesterol, glucose, and triglycerides levels in animal models of diabetes [57]

To further identify the target proteins of B-TCE-mediated suppression of lipogenesis, the expression of key molecules regulating fatty acid, sterol, and triglyceride synthesis were studied. B-TCE-supplemented sleep-deprived animals showed an increase in the phosphorylated form of AMPK and its substrate ACC in TSD and TUD groups as compared to the VSD group (Fig. 2d, e). AMPK is a serine/threonine kinase that is activated during stress conditions and functions to regulate glucose and lipid metabolism in the liver by phosphorylating downstream molecules such as ACC, SREBP-1, and HMG-CoAR [58]. ACC catalyzes the conversion of acetyl CoA to malonyl CoA, an inhibitor of carnitine palmitoyl transferase 1 (CPT1) which is essential for the entry of fatty acids into mitochondria for β-oxidation. However, phosphorylation of ACC at Ser-79 by pAMPK inhibits its catalytic activity [59]. Phosphorylation of ACC was downregulated in the sleep-deprived VSD group and was upregulated in the B-TCE pre-treatment (Fig. 2d, e), thus suggesting that some active ingredients of B-TCE targeted AMPK and its downstream pathway to promote fatty acid oxidation in these sleep-deprived animals.

Acute sleep deprivation-induced fatty acid and triglyceride synthesis in the liver as evident from upregulated expression of SREBP-1 and FAS in the VSD group, was suppressed in the B-TCE pre-treated TSD and TUD group animals (Fig. 3). These findings are also supported by the reduced serum levels of triglycerides and cholesterol of TSD and TUD group animals. SREBP-1 is involved in triglyceride synthesis by activating the lipogenesis gene, FAS [60]. Upregulation of hepatic lipogenic enzymes has been reported to cause SD-induced hepatic steatosis and insulin resistance [55]. HMGCoAR is the third enzyme of the cholesterol synthesis pathway that converts HMG-CoA to mevalonate using two molecules of NADPH [61]. It is under strict regulation at the transcriptional, translational, and post-translational levels and is inactivated on phosphorylation. AMPK is the principal kinase that is responsible for its inactivation [50]. Decrease in the expression of HMG-CoAR, and serum cholesterol levels in TSD and TUD groups may suggest that active ingredients present in B-TCE targeted at multiple levels to ameliorate dyslipidemia induced by SD. Similarly, the downregulation of lipolysis regulating enzyme ATGL expression and increase in serum triglycerides by sleep deprivation was normalized by B-TCE in TSD group animals (Fig. 3). Dyslipidemia in post-menopausal women is also attributed to reduced expression of lipolysis-related lipases [62]. Hepatic ATGL is thought to be involved in triglyceride routing and partitioning, either by enhancing free fatty acid release and oxidation or by promoting VLDL synthesis [63]. B-TCE induced a decrease in triglyceride accumulation in TSD and TUD group animals along with upregulation of ATGL expression suggesting that the extract induced lipolysis by enhancing lipase activity and simultaneously inhibited lipogenesis by targeting triglyceride biosynthesis regulatory proteins. Taken together, these results suggest that B-TCE due to its multiple active ingredients targeted the oxidation of fatty acids as well as inhibited cholesterol and triglyceride biosynthesis by activating AMPK and its downstream signaling molecules of lipid metabolism.

PI3K/Akt signaling pathway is known to regulate cell growth, proliferation, migration, and survival in the liver [64]. p-Akt expression was significantly downregulated in the VSD group which was normalized in the TSD and TUD groups suggesting activation of p-Akt by B-TCE (Fig. 4). Upon activation, Akt regulates the activity of CyclinD1 and Bad, although by different pathways thus promoting cell proliferation and survival [65, 66]. Activated Akt also leads to nuclear accumulation of CyclinD1, a cell cycle regulatory protein thus resulting in cell proliferation [65]. CyclinD1 expression was significantly downregulated in the VSD group which was normalized by B-TCE supplementation in TSD and TUD groups (Fig. 4). Bad, a pro-apoptotic protein is another downstream target of Akt signaling [67]. Under normal conditions, Bad protein translocates from cytoplasm to mitochondria where it associates with an anti-apoptotic protein, Bcl-xL leading to its inactivation and promoting mitochondrial dysfunction and apoptosis. This translocation is inhibited by phosphorylating Bad at Ser-136, leading to its cytosolic sequestration [68]. The current data provide evidence that SD inhibited phosphorylation of Bad in the VSD group which was prevented by pre-treatment with B-TCE before SD (Fig. 4). In addition, Bad phosphorylation by B-TCE was also accompanied by anti-apoptotic protein, Bcl-xL activation. The decrease in the expression of proapoptotic marker AP1 in B-TCE pre-treated TSD and TUD groups further supports the cell survival-promoting activity of B-TCE (Fig. 5b). SD is a known risk factor for CVDs, especially in the elderly population which results in impaired endothelial function [69]. e-NOS plays an important role in cardiovascular functions by regulating the synthesis of nitric oxide. Activated Akt regulates e-NOS expression to maintain the normal functioning of the endothelial cells which on inhibition causes inflammation and hypoxia [70]. SD reduced e-NOS expression was normalized in TSD and TUD groups thus suggesting that B-TCE also targeted e-NOS to maintain normal endothelial functions in the liver tissue (Fig. 5c).

## Conclusions

Poor sleep quality in perimenopausal/postmenopausal women is associated with several metabolic and cardiovascular disorders in women in their later life. Therefore, investigating sleep disturbances and related pathologies during the menopause transition phase of women may help to develop effective interventions for improving their health and quality of life. The current findings suggest that this indigenous medicinal plant extract B-TCE due to its multi-component nature and targeting multiple pathways may be used to develop a herbal-based concoction for the management of menopause-associated deterioration of sleep quality and associated metabolic disorders. However, there are certain limitations of the current study which will be addressed in our future study with this medicinal plant. Our next endeavour is to identify the active compounds responsible for the hepatoprotection imparted by B-TCE using appropriate analytical techniques. Further to confirm the role of AMPK and its downstream lipid metabolism pathways as well as the p-Akt/cyclinD1/p-bad pathway in mediating hepatoprotection, experiments will be designed with pathway-specific inhibitors.

## Data Availability

All data generated or analysed during this study are included in this published article.

## Abbreviations

ACC: Acetyl-CoA carboxylase
Akt: Protein kinase B
ALP: Alkaline phosphatase
ALT: Alanine transaminase
AMPK: AMP-activated protein kinase
AST: Aspartate aminotransferase
ATGL: Adipose triglyceride lipase
Bad: Bcl-2 associated death promoter
B-TCE: Butanol extract of *T. cordifolia*
CPT1: Carnitine palmitoyl transferase 1
e-NOS: Endothelial nitric oxide synthase
FAS: Fatty acid synthase
GSH: Glutathione
HDL: High-density lipoproteins
HMG-CoAR: 3-Hydroxy-3-methylglutaryl-coenzyme A
HPG axis: Hypothalamic-Pituitary–Gonadal axis
KCl: Potassium chloride
LDL-C: Low-density lipoprotein cholesterol
ROS: Reactive oxygen species
SAG: Superoxide anion generation
SREBP-1: Sterol regulatory element-binding protein 1c
TBARS: Thiobarbituric acid reactive substances
TCE: 50% Ethanolic extract
TCP: *T. cordifolia* Stem powder
TSD: B-TCE pre-treated sleep-deprived
TUD: B-TCE pre-treated undisturbed sleep
VSD: Vehicle-sleep deprived
VUD: Vehicle-undisturbed
